# Mid-upper arm circumference as a substitute for body mass index in the assessment of nutritional status among adults in eastern Sudan

**DOI:** 10.1186/s12889-022-14536-4

**Published:** 2022-11-10

**Authors:** Imad R. Musa, Saeed M. Omar, Ishag Adam

**Affiliations:** 1 Royal Commission Hospital at AL Jubail Industrial City, Al Jubail, Kingdom of Saudi Arabia; 2 Faculty of Medicine, Gadarif University, Gadarif, Sudan; 3 Department of Obstetrics and Gynecology, Unaizah College of Medicine and Medical Sciences, Qassim University, Unaizah, Kingdom of Saudi Arabia

**Keywords:** Anthropometry, Body mass index, Mid-upper arm circumference, Underweight, Obesity, Sudanese adults

## Abstract

**Background:**

Body mass index (BMI) remains the most used indicator of nutritional status despite the presence of a potentially credible alternative. Mid-upper arm circumference (MUAC) is an anthropometric measure that requires simple equipment and minimal training. The aim of this study was to compare MUAC with BMI and propose a MUAC cut-off point corresponding to a BMI of < 18.5 kg/m^2^ (underweight) and ≥ 30.0 kg/m^2^ (obesity) among Sudanese adults.

**Methods:**

A cross-sectional study using multistage cluster sampling was conducted in New-Halfa, eastern Sudan. Participants’ age and sex were recorded and their MUAC, weight and height were measured using the standard procedures. The MUAC (cm) cut-offs corresponding to < 18.5 kg/m^2^ and ≥ 30.0 kg/m^2^ were calculated and determined using receiver operating characteristic (ROC) curve analysis

**Results:**

Five hundreds and fifty-two adults were enrolled in the study. The median (interquartile range, IQR) of the participants age was 31.0 (24.0 – 40.0) years and 331 (60.0%) of them were females. The medians (IQR) of BMI and MUAC were 22.4 (19.1 – 26.3) kg/m^2^ and 25.0 (23.0 – 28.0) cm, respectively.

There was a significant positive correlation between MUAC and BMI (r = 0.673, *p* < 0.001).

Of the 552 enrolled participants, 104 (18.8%), 282 (51.1%), 89 (16.1%) and 77 (13.9%) were normal weight, underweight, overweight and obese, respectively. Best statistically derived MUAC cut-off corresponding to a BMI < 18.5 kg/m^2^ (underweight) was ≤ 25.5 cm in both males and females (Youden’s Index, YI = 0.51; sensitivity = 96.0%; specificity = 54.0%), with a good predictive value (AUROCC = 0.82). Best statistically derived MUAC cut-off corresponding to a BMI ≥ 30.0 kg/m^2^ (obesity) was ≥ 29.5 cm in both males and females (YI = 0.62, sensitivity = 70.3%, specificity = 92.0%), with a good predictive value (AUROCC = 0.86, 95.0% CI = 0.76 – 0.95).

**Conclusion:**

The results suggest that the cut-offs based on MUAC can be used for community-based screening of underweight and obesity

## Introduction

Nutrition-related medical problems are common public health issues with high risks of mortality and economic loss, especially in the developing countries [1]. Body weight and height are used to compute body mass index (BMI), which is widely used to assess nutritional status in adults [2, 3]. BMI is an indicator of malnutrition with an individual being considered underweight when BMI is < 18.5 kg/m^2^ [4]. Undernutrition affects the function and recovery of almost all body organs [5]. On the other hand, BMI is also utilised to diagnose obesity [4]. Obesity (BMI ≥ 30 kg/m^2^) represents a threat that increases the risk of several non-communicable diseases [4, 6]. It is associated with elements of metabolic syndrome and its complications, cancer, kidney disease, mental/central nervous system diseases, as well as musculoskeletal, respiratory, reproductive and dermatological disorders [7]. The nutritional status problems in adults are neglected or underestimated in developing countries [1]. Data from sub-Saharan Africa, highlight this health problem, as up to half (6.0 – 48.0%) of elderly Africans are underweight, almost a quarter (2.5 – 21.0%) are overweight and 56.0% of older South Africans are obese [8].

BMI may be biased by fluid overload and oedemas, and therefore does not reflect body composition; for example, a high BMI can be seen in fat people and also in very muscular athletes [3, 9, 10]. The simplicity of MUAC measurement means it does not require mathematical derivation [11], frontline professionals or expensive equipment compared to the requirements for measuring BMI [11]. MUAC is a practical tool for screening and grading the severity of nutritional status problems in the general population [12, 13] and it has demonstrated superiority over BMI [6]. Moreover, previous studies have reported a positive correlation between MUAC and BMI [11, 14]. Several studies have identified the MUAC cut-off measurement as an alternative method of detecting underweight [11, 13–15] and obesity [16, 17].

Nutritional health problems are significant in Sudan [18, 19], but there are no published data that evaluate the use of MUAC measurement among adults to detect their nutritional status. Obtaining specific MUAC cut-off for certain areas of the population can be an important method in developing countries in general and particularly in Sudan. The present study was conducted to evaluate the use of MUAC as a reliable alternative to BMI for detecting malnutrition and obesity among adult Sudanese in New Halfa.

## Methods

### The setting, subjects and data collection

The Strengthening the Reporting of Observational studies in Epidemiology (STROBE) Statement standard checklists were followed [20]. A multistage sampling study was adopted in New Halfa, eastern Sudan. Initially, four sectors (lowest administrative unit) out of the seven sectors were selected by simple random sampling. The total sample size of 552 (please see below) participants was distributed across the four sectors according to the size allocation of the sector. Then, all the agreed adults (18 – 60 years of age) healthy Sudanese participants from the household were then selected using a lottery method. When a selected house was not inhabited or the inhabitants refused to participate or had met one of the exclusion criteria, the next house was chosen. Trained general practitioners interviewed the participants during the period of January to February 2022.

### Inclusion criteria

- A multistage sampling method for selection.
- Apparently healthy males and females.
- Participants who were residents of New Halfa.
- Age (≥ 18 years – ≤ 60 years).
- Signed consent for participation.

### Exclusion criteria

- Age below 18 years and above 60 years.
- Pregnant women.
- Individuals with chronic diseases such as diabetes, thyroid diseases and heart failure.
- Critically ill patients with severe acute illness.
- Athletes, persons with a mental illness or disability, hormonal or any apparent congenital dysmorphism.
- Individuals on chronic medications
- Individuals who refused to participate.

Based on our selection criteria, the eligible participants were approached between 9 am and 4 pm from Sunday to Thursday. Age, sex, weight (to the nearest 10.0 g), standing height (to the nearest 1mm) and MUAC (to the nearest 1mm) in sitting or standing posture were measured following standard procedures using calibrated instruments. During weight measurement, each subject was asked to stand after removing their shoes, any heavy clothing and any objects in their pockets. They were then instructed to relax with their arms at the sides and feet positioned close together to evenly distribute their weight across their feet. Height was measured by a stadiometer without shoes and socks, while the feet were positioned together at the heels to ensure the back of the heels, buttocks and shoulder blades touched the back plate/stick. Besides this, the head was adjusted in the Frankfurt horizontal plane. The MUAC was measured using a non-stretchable MUAC measuring tape. The tape was placed at a point equidistant between the acromion process of the left scapula and the olecranon process of the left ulna to measure the MUAC. All anthropometric measurements were performed twice, and the average was recorded. A third measurement was obtained in case of considerable variation between the first two measurements (differences of more than 100.0 g for weight, 0.5 cm for height and 0.2 cm for MUAC). The average of the nearest two measurements was recorded. BMI was calculated using the standard formula: weight in kg/height in m^2^ [4]. In accordance with the WHO criteria, the BMI cut-off points of < 18.5kg/m ^2^ and ≥ 30.0 kg/m^2^ were used to identify adults who were underweight and those with obesity, respectively [4].

### Determining sensitivity and specificity

Sensitivity and specificity are of diagnostically equal importance; hence the Youden index (YI) was used to indicate the best performance (the larger the better) at a given cut-off. The Statistical Package for the Social Sciences (SPSS) was used to obtain the receiver operating characteristic (ROC) curve and coordinates of the curve that indicate sensitivity and 1−specificity. Then the *J*-index was calculated as the YI = sensitivity + specificity - 1 to locate the suitable cut-off points depending on the highest *J*-index. Then we suggested the best cut-off point based on the highest value of YI to show the best sensitivity and specificity.

### Sample size calculation

A sample of 552 participants adults was calculated to have the significant minimum difference in the correlations (*r* = 0.15) for calculated BMI and measured MUAC, This sample (552 adults) would have an 80% power and a difference of 5% at *α* = 0.0 5[21]. To ensure state-wide representativeness, respondents were selected from all sectors after New Halfa was divided in to four sectors according to its population.

### Statistical analysis

Data were collected and statistical analyses were performed using IBM SPSS v.25. Normality in distribution was tested using Shapiro-Wilk tests. The data were not normally distributed hence Mann-Whitney U was used to test the difference of variables between males and females. Descriptive statistics were generated for demographic variables, all measurements (weight, height and MUAC) and BMI. Scatterplots with fitted linear regression lines were calculated to assess the relationship between MUAC and BMI, and correlation analysis was performed using Spearman’s correlation to obtain correlation coefficients and P value for all females and male participants, Sensitivity and specificity were calculated for all individual measurements in the dataset. Youden’s Index (YI) was calculated as YI = sensitivity + specificity - 1. The MUAC cut-off with the highest YI-value was considered the optimal statistically-derived cut-off [22]. The area under the receiver operating characteristic curve (AUROCC) was calculated for all participants and females and male separately. *P* value less than 0.05 was adopted for significance.

## Results

Five hundreds and fifty-two participants were enrolled in the study. The median (IQR) of the age was 31.0 (24.0 – 40.0) years and 331 (60.0%) of them were females. The medians (IQR) of BMI and MUAC were 22.4 (19.1 – 26.3) kg/m^2^ and 25.0 (23.0 – 28.0) cm, respectively.

**Fig 1:**
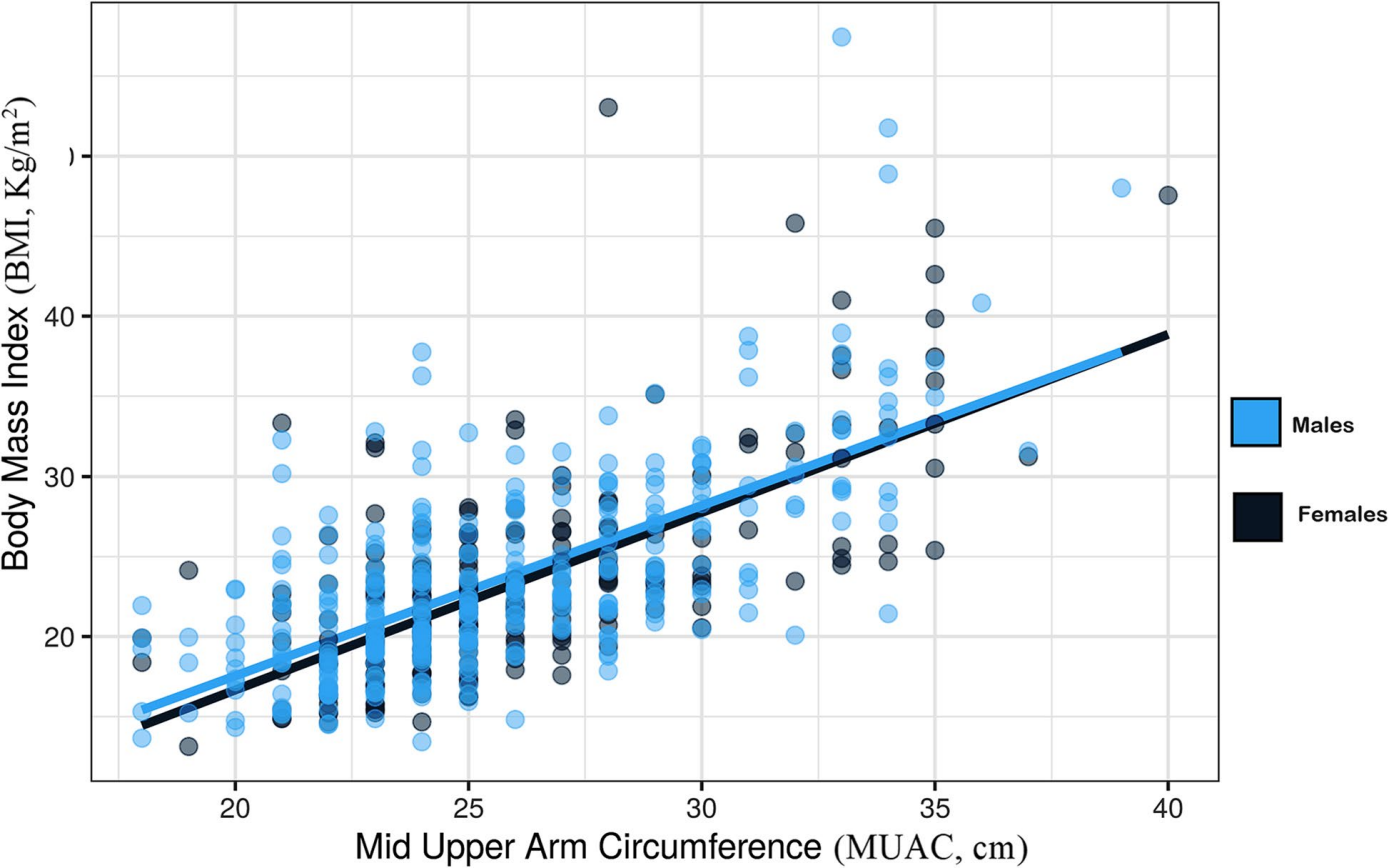
Curve estimation for assessing linear relationship between mid-upper arm circumference and body mass index in adults in eastern Sudan 2022

While males were significantly elder and taller, for weight, BMI and MUAC there were no differences between males and females, Table 1. There was a significant positive correlation between MUAC and BMI (*r* = 0.673, *p* < 0.001) in both females and males, in females (*r* = 0.639, p < 0.001) and in males (*r* = 0.634, *p* < 0.001), Fig. 1.

**Table 1:** Comparison of anthropometric profile between men and women from eastern Sudan

	Total (number =552)	Female (number =331)	Male (number =221)	P
Age, years.	31.0 (24.0 – 40.0)	30.0 (24.0 – 39.0)	33.0 (26.0 – 44.0)	0.040
Weight, kg	58.2 (46.0 – 70.0)	56.0 (45.0 – 70.0)	59.0 (48.0 – 70.0)	0.127
Height, cm	160.0 (150.0 – 170.0)	160.0 (150.0 – 168.0)	163.0 (154.0 – 172.0)	<0.001
Body mass index, kg/m^2^.	22.4 (19.1 – 26.3)	22.31 (19.2 – 27.01)	22.4 (18.8 –25.2)	0.437
Mid upper arm circumference, cm	25.0 (23.0 – 28.0)	25.0 (23.0 – 28.0)	25.0 (23.0 – 29.0)	0.439

Of the 552 enrolled participants, 104 (18.8%), 282 (51.1%), 89 (16.1%) and 77(13.9%) were normal weight, underweight, overweight, and obese, respectively.

**Fig. 2:**
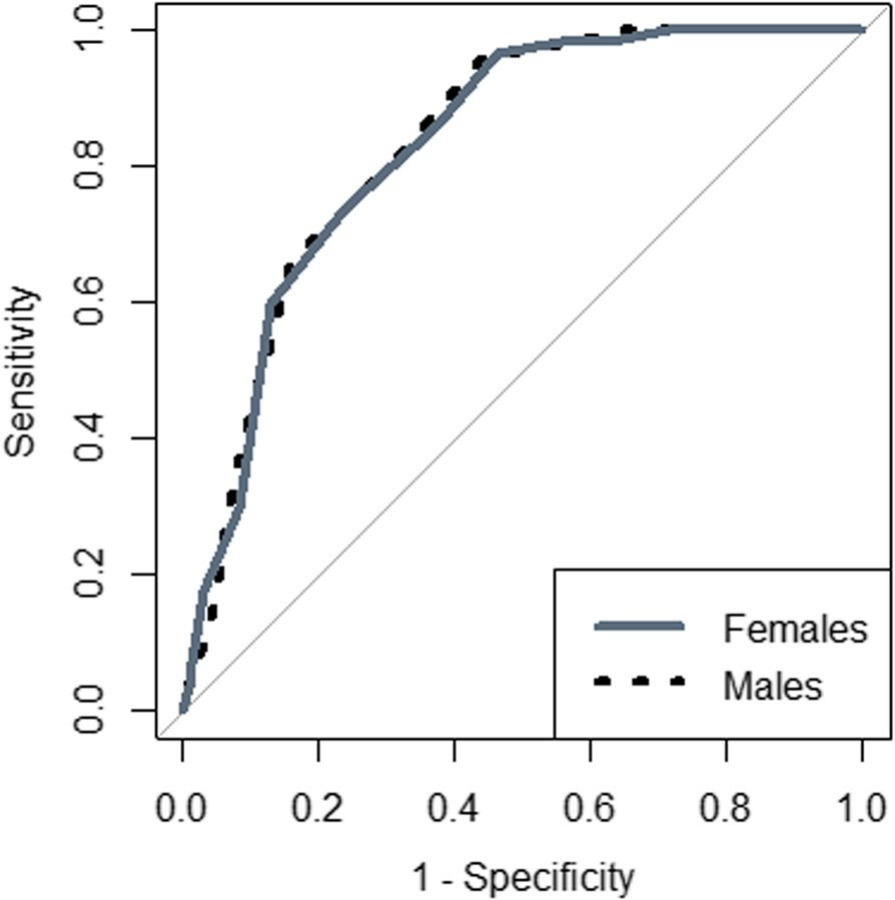
Receiver operating curve of mid-upper-arm-circumference for underweight (body mass index <18.5 kg/m^2^) for adults in eastern Sudan 2022

The best statistically derived MUAC cut-off corresponding to a BMI < 18.5 kg/m^2^ (underweight) was ≤ 25.5 cm in both males and females (YI = 0.51; sensitivity = 96.0%; specificity = 54.0%), with a good predictive value (AUROCC = 0.82, 95.0% CI = 0.78 – 0.86), in females (YI = 0.51, sensitivity = 97.0%, specificity = 54.0%), with a good predictive value (AUROCC = 0.83, 95.0% CI = 0.78 – 0.87) and in males (YI = 0.51, sensitivity = 96.0%, specificity = 56.0%), with a good predictive value (AUROCC = 0.83,95.0% CI = 0.77 – 0.88) (Table 2 and Fig. 2).

**Table 2:** MUAC cut-off points for the diagnosis of underweight and obesity in adults from eastern Sudan, 2022

	Underweight (body mass index<18.5 kg/m^2^)			Obese (body mass index≥ 30.0 kg/m^2^)		
	All participants	Females	Males	All participants	Females	Males
Mid-upper arm circumference cut-off	≤ 25.5 cm	≤ 25.5 cm	≤ 25.5 cm	≥ 29.5 cm	≥ 29.5 cm	≥ 29.5 cm
Area under the curve (95.0% confidence interval)	0.82 (0.78–0.86)	0.83 (0.78–0.87)	0.83 (0.77–0.88)	0.84 (0.78–0.90)	0.84 (0.76–0.91)	0.86 (0.76–0.95)
Youden's index	0.51	0.51	0.51	0.62	0.59	0.64
Sensitivity	96.0	97.0	96.0	70.3	68.9	72.4
Specificity	54.0	54.0	56.0	92.0	91.0	92.2

**Fig. 3:**
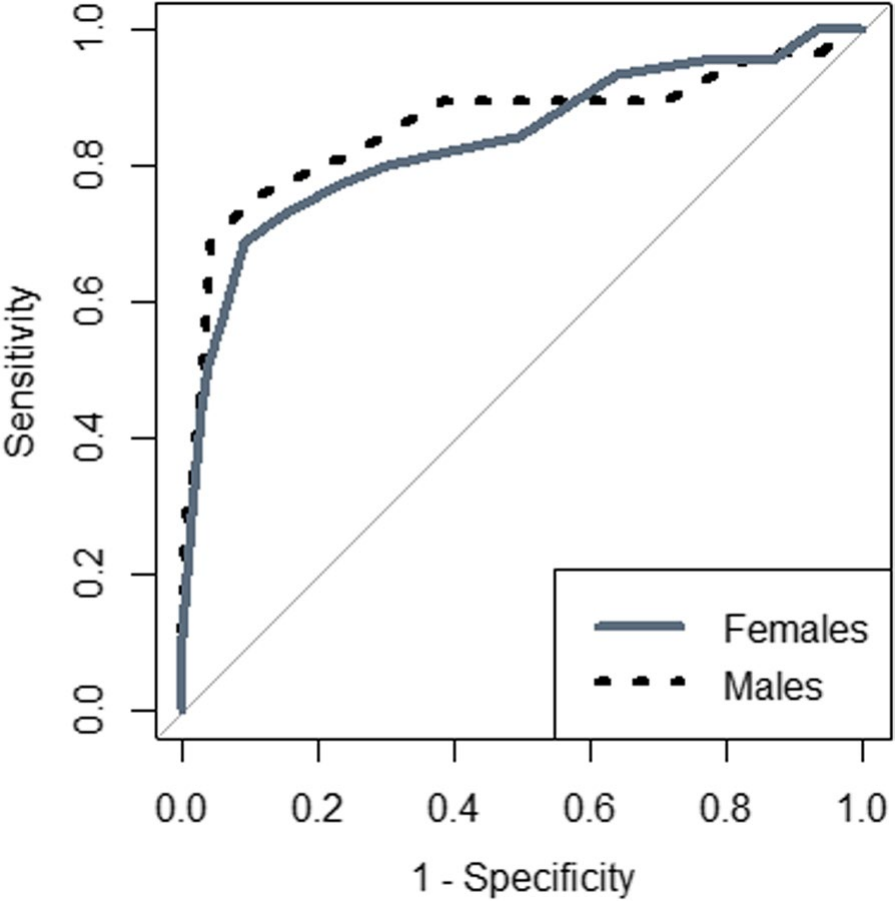
Receiver operating curve of mid-upper-arm-circumference for obesity t (body mass index ≥ 30.0 kg/m^2^) for adults in eastern Sudan 2022

The best statistically derived MUAC cut-off corresponding to a BMI ≥ 30.0 kg/m^2^ (obesity) was ≥ 29.5 cm in both males and females (Y I = 0.62, sensitivity = 70.3%, specificity = 92.0%), with a good predictive value (AUROCC = 0.84, 95.0% CI = 0.78–0.90), in females (YI = 0.59, sensitivity = 68.9%, specificity = 91.0%), with a good predictive value (AUROCC = 0.84, 95.0 % CI = 0.76–0.91) and in males (YI = 0.64, sensitivity = 72.4%, specificity = 92.2%), with a good predictive value (AUROCC = 0.86, 95.0 CI = 0.76 – 0.95) (Table 2 and Fig. 3).

## Discussion

The current study demonstrated a positive correlation between BMI and MUAC in all participants and in male and non-pregnant female adults separately. This was similar to the previous findings in several studies, e.g., in South Sudan [16, 23], Bangladesh (males *r* = 0.81, versus females *r* = 0.828,) [11], Nepal (women *r* = 0.889 and men *r* = 0.846) [14], Spain (*r* = 0.78) [24] and in India (*r* = 0.81) [25].

In the current study a statistically derived MUAC cut-off for an underweight individual was ≤ 25.5 cm, which showed similar results in females and males (YI = 0.51; sensitivity = 96.0%; specificity = 54.0% and AUROCC = 0.82). The MUAC cut-off proposed in our study to detect underweight, was similar to that obtained recently in South Sudan (MUAC ≤ 25.4 cm, sensitivity = 87.0% and specificity = 81.0%) [16] and among adults in Sub-Saharan African countries (≤ 25·5cm, sensitivity = 77.0% and specificity = 79.6%) [26]. Moreover, the same cut-off of MUAC (≤ 25.5 cm) was adopted to assess nutritional risk, three months post-acute stroke, in patients aged over 65 years in South-eastern Norway [27].

Furthermore, MUAC cut-off to detect underweight, in the current study, was slighter higher than that reported in a meta-analysis study, evaluating twenty datasets from Africa (13,835 participants) (≤ 25·0 cm) [15], female and male farmers in Tanzania and Mozambique (≤ 25.0 cm) [13], in Nepal (≤ 24.5 cm, sensitivity = 92.8%, specificity = 82.4% and AUROCC > 0.9) [14] and in Bangladesh (≤25.1 cm, = AUROCC 0.93) [11]. Additionally, MUAC cut-off < 24.0 cm was reported among adults in different studies across the globe such as in South Africa (sensitivity = 89.3% and specificity= 82.9%) [12], in Kolkata, India (male ≤ 24.0 cm) [28, 29], in eastern India (males ≤ 22·7 cm and female ≤ 21·9 cm) [30], in northern Vietnam (≤ 23.5 cm) [17], and in females in Bangladesh (≤ 23.9 cm) [11]. Likewise, a markedly lower MUAC cut -off was reported from another study that recruited 2,421 men and 3,248 women from selected regions of five African countries, India, China and Papua New Guinea: (≤23.0 cm in male and ≤22.0 cm in female) [31] as well as in Spain (22.5 cm) [24]. The summary of the different cut-off points which were reported in several previous studies across the globe are shown in Table 3.

**Table 3:** MUAC cut-off points for the identification of underweight and obesity in various populations around the world

Author	Year	Country	Total number	Males (N)	Females (N)	MUAC cut -off, cm	Youden’s index	sensitivity	specificity	AUROCC	r
**underweight**											
Tang et al.	2020	Different countries	13835	4860	8993	24	--	84 %	83 %	--	--
Thorup et al.	2020	Nepal	302	105	197	24.5	0.75	92.86 %	82.48 %	> 0.9	F;.889 M;.846.
Sultana et al.	2015	Bangladesh	650	260	390	F <24 M <25	F; 0.69 M; 0.72	F; 92.6 % M;92.6 %	F;76.46 % M;79.6 %	F;0.93 M; 0.93	F;.828 M = 0.8
Eleraky et al.	2021	Tanzania and Mozambique	2106	689	1417	<25	0.67	77.8 %	87.2 %	--	0.837
Chakraborty et al.	2011	India	205	205		24	0.45	74.8 %	70.3 %	0.79	0.45
Chakraborty et al.	2009	India	474	474		24	--				0.84
Amegovu et al.	2020	South Sudan	251	214	37	< 25.4	--	87 %	81 %	90%	0.8621
Nguyen et al.	2014	Vietnam	4,981		4,981	23.5	--	89.1 %	71 %	0.905	0.84
Tonder et al.	2019	multi-centre	266	138	128	23.7	--	89.3 %	82.9 %	0.92	0.93,
Das et al.	2020	India	618	--	618	23.2	--	89 %	82 %	0.923	0.81
Das et al.	2018	India	955	467	488	M;22·7 F;21·9	M;0·605 F; 0·72	M;85.71 % F;91.67 %	M;74·80 % F;79.89 %	M;0·85 F;0·93	0·71
Philpott et al.	2021	sub-Saharan	11 917	11 917	--	25·5	---	77 %	79·6 % %	0·87	--
Benítez Brito et al	2016	Spain	1373	778	595	≤22.5	--	67.7 %	94.5 %	0.92	0.78
**Obesity**											
Amegovu et al.	2020	South Sudan	251	214	37	>31.1	--	100 %	96 %	.99	0.8621
Das et al.	2020	India	618		618	30.5	--	99 %	83 %	0.876	0.81
Tonder et al.	2019	South Africa	266	138	128	29.4	--	100 %	87.2 %	0.97	--
Eleraky et al..	2021	Tanzania and Mozambique	2106	689	1417	> 31.5	0.88	95.8	92.1	--	0.837
Shifraw T et al.	2021	Ethiopia	4914	-	4914	>30.0	0.7	87.0	83.0	0.93	

*MUAC* Mid upper arm circumference, *AUROCC* Area Under the Receiver Operating Characteristics Curve, r; Correlation coefficient

In the current study, a statistically derived MUAC cut-off for underweight was ≥ 29.5 cm, which was similar in females and males (YI = 0.60; sensitivity = 69.0% and specificity = 92.0%). A similar MUAC cut-off (> 29.4 cm, sensitivity = 100% and specificity = 87.2%) was proposed and obtained in South Africa [12]. On the other hand, slightly higher MUAC cut-offs were demonstrated in the neighbouring country, South Sudan (31.1cm, sensitivity = 100% and specificity = 96.0%) [16], in Ethiopia (≥ 30.0 cm, sensitivity = 87.0 % and specificity = 83.0%) [32], among female and male farmers in Tanzania and Mozambique (≥ 31.5 cm ) [13], in northern Vietnam (≥ 31cm) [17] and among Chinese males (≥ 30.9 cm) [33].

While one study questioned the diagnostic accuracy of BMI when diagnosing obesity and found it to be limited, particularly for individuals in the intermediate BMI ranges [9]. Another study recommended using BMI to detect only overweight related problems [10]. Furthermore, another study concluded that BMI was not a reliable measurement of body composition in individuals, in particular older and younger individuals [34].

We compare our results with others’ findings with caution because we (and some others) determined the cut-off levels for MUAC using the equation YI. It is believed that optimal cut-off should not be determined strictly based on YI, but instead by judging and considering the balance between high sensitivity and high specificity. Moreover, many contributing factors may explain the variation in MUAC cut-off for nutritional status in different studies and populations: the previously documented racial/ethnic issues creating variable cut-offs [12, 28, 35]. Hence, ethno-specific cut-offs for MUAC are proposed to assess the nutritional status for certain populations. While the current study recorded no gender variation regarding MUAC cut off, several previous studies reported MUAC cut-off variation for nutritional status [33, 36]. This could be explained by sex fat distribution during aging, as males tend to have more visceral fat in the abdomen (apple shape), and females tend to have more subcutaneous fat in the hip and thighs (pear shape) before menopause [37]. The main advantages of MUAC method compared to BMI method are the simplicity of MUAC measurement, which does not require mathematical derivation [11], frontline professionals (medical or paramedical staff) [12] and requires relativity low -cost equipment [11, 25], besides being an effective measure of screening for poor nutritional status in adults [38] and during famine and emergencies [23, 39]. MUAC emerges as a useful, applicable and alterative measure of nutritional status in nearly all acutely ill patients whom measurements of weight and height may be inappropriate or impossible [40] and among those who have medical conditions affecting the BMI: the localized accumulation of excess fluid (oedema, ascites) [41]. Hence, measurement of MUAC, is a simple non-invasive method, can be used to assess nutritional status in adult in remote area or alternative method for BMI especially in setting where BMI cannot be used (oedema and immobile patients).

One limitation of the cut-off proposed in the present study is that the data collected from one source location which may not totally represent the entire population in Sudan. Moreover, it was a relatively small sample size. As procedure like BMI, for estimating nutritional status, always has its limitations in specific populations e.g., athletes. A higher sample size might have increased the prevalence of the outcome, which again could increase the strength of the analysis.

## Conclusion

Our study proposes the cut-offs based on MUAC (≤ 25.5 cm and ≥ 29.5 cm) as alternative for BMI for community-based screening of underweight and obesity, respectively.

## Data Availability

The datasets generated and/or analyzed during the current study are not publicly available (because the manuscript is still under the peer review process) but are available from the corresponding author on reasonable request.

## Abbreviations

kg: kilogram
m: meter
cm: centimeter
BMI: Body mass index
CI: Confidence interval
ROC: receiver operating characteristic curve
AUROCC: Area Under the Receiver Operating Characteristics Curve
YI: Youden’s Index
SPSS: Statistical Package for the Social Sciences
IQR: Interquartile range
STROBE: The Strengthening the Reporting of Observational studies in Epidemiology
